# 6.L. Round table: Digitally literate health workforce for a resilient future

**DOI:** 10.1093/eurpub/ckac129.383

**Published:** 2022-10-25

**Authors:** Organised by: Young Forum Gastein, EUPHAnxt, Chair persons: Matthias Wismar (European Observatory)

## Abstract

Currently, health systems worldwide are experiencing an unprecedented challenge after the shock caused by the COVID-19 pandemic. However, there lies an opportunity for renewal to strengthen health systems. The importance of collaboration becomes imperative in times of health workforce (HWF) shortages and demanding working conditions. This workshop, organised in partnership by the Young Forum Gastein and EUPHAnxt, will focus on connecting generations to explore, discover and share best practices for HWF education and (re)training in digital skills to deliver better patient care. The COVID-19 pandemic has accelerated digitalisation across many sectors including healthcare. Yet it has also served to highlight the digital divide, which has the potential to hinder health experts in providing optimal care. Concurrently, the demanding working conditions of the HWF can be improved by making use of digital tools. It is high time to discuss how digital literacy can be improved across a multi-generational HWF and how to empower the next generation of healthcare leaders to re-imagine health services. The workshop will bring different perspectives to the table - policy-making, science & academia, and governance - on the approaches for better intergenerational collaboration by making use of digital literacy. At the end of the session, participants will understand the realistic potential of digital literacy and how it can be used to ensure effective communication across the HWF, support the resilience of health systems, and ultimately, safeguarding patient care.

**Key messages:**

• Digital literacy is not a stable level of knowledge - the health workforce needs opportunities to advance their knowledge and (digital) skills.

• Acknowledging the shortages in health workforce planning, it is essential to ensure adequate skills of the health workforce personnel.

**Speakers/Panellists:**

**Natasha Azzopardi Muscat**

WHO Regional Office for Europe, Copenhagen, Denmark

**Marius-Ionut Ungureanu**

Department of Public Health of the Babeș-Bolyai University, Cluj-Napoca, Romania

**Brian Wong**

EUPHA-DH

GHFutures2030, I-DAIR, London, UK

**Ellen Kuhlmann**

Medizinische Hochschule Hannover, Hannover, Germany

